# Parosteal lipoma of the proximal humerus mimicking atypical lipomatous tumor: A diagnostic challenge

**DOI:** 10.1016/j.radcr.2025.08.083

**Published:** 2025-09-15

**Authors:** Cameron Nosrat, Kevin Sweetwood, Daria Motamedi

**Affiliations:** Department of Radiology, University of California, 513 Parnassus Ave, Room S257, Box 0628, San Francisco, CA 94143, USA

## Abstract

Parosteal lipomas are a rare benign fat-containing neoplasm that originates from mature adipose tissue near the bone periosteum that can often be difficult to differentiate from malignant tumors. We present the case of a 75-year-old female presenting with several months of a growing, uncomfortable right shoulder mass without neurological or motor deficits. Computerized tomography (CT) demonstrated a well-defined mass located deep to the deltoid along the proximal humerus with peripheral fat attenuation while follow-up magnetic resonance imaging (MRI) demonstrated internal central enhancement and thin fibrous septae. Such findings were most consistent with a parosteal lipoma, and given the indolent nature of the mass, absence of aggressive features, and stable size over a 5-month period, conservative management with observation and interval imaging was recommended. This case emphasizes that while histological confirmation remains the gold standard, magnetic resonance imaging (MRI) is currently the most valuable tool in differentiating parosteal lipoma from atypical lipomatous tumors and other fat-containing lesions and guiding management decisions.

## Introduction

While lipomas are the most common benign soft tissue tumors commonly appearing in the subcutaneous tissues, deep lipomas are extremely uncommon, occasionally causing symptoms when seated near a nerve or blood vessel [1]. These deep-seated lipomas can be characterized as inter or intramuscular, infiltrative, and parosteal [2]. Parosteal lipomas are a rare benign fat-containing neoplasm that originates from mature adipose tissue near the bone periosteum [1,[3], [4], [5]]. They account for 0.3% of all lipomas, traditionally localizing to the diaphysis of the femur and proximal radius [1,[3], [4], [5]]. Lesions typically show no sex preference and manifest in individuals between 40 and 60 years of age [6]. These lesions are typically associated with periosteal changes similar to those seen malignant tumors, such as liposarcoma and malignant fibrous histiocytoma, making imaging in conjunction with pathology important for diagnosis [7]. The origin of parosteal lipomas is thought to involve either aberrant differentiation of mesenchymal stem cells within adipose tissue or a reactive transformation of fibroblasts, potentially caused by chronic trauma, metabolic imbalance, or localized ischemia [8,9]. There currently is no method for confirming the diagnosis preoperatively.

## Case report

This study reports the case of 75-year-old female with a past medical history of hypothyroidism who presented to clinic for evaluation of a right shoulder mass. The patient first noticed the mass approximately 5 months prior and reported that it had gradually increased in size. She denied any trauma to the area. The mass was not painful at rest, but she described discomfort with certain shoulder movements and when lying on the affected side. She denied subjective weakness or limitations in range of motion. Notably, she had a prior history of schwannoma resection and a longstanding thyroid-associated mass that had been biopsied multiple times without conclusive results.

On physical examination, the mass was firm, non-mobile, and palpable over the anterior portion of the right shoulder without overlying skin changes. Neurologic and motor function of the right upper extremity was intact, including 5/5 strength in deltoid, biceps, triceps, wrist extensors, and finger flexors. Sensation was preserved, and there were no vascular or skin abnormalities.

**Fig. 1:**
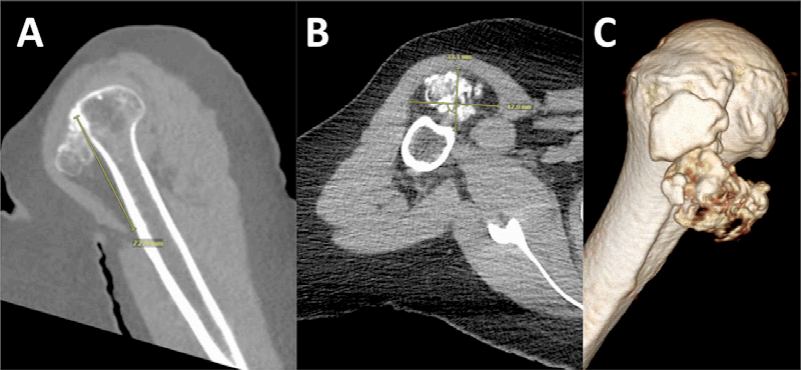
(A) Coronal, (B) Axial CT, and (C) 3D Reconstruction helical axial acquisitions encompassing the right shoulder without intravenous contrast.

Initial evaluation with computerized tomography (CT) of the upper extremity demonstrated a well-defined mass located along the anterior aspect of the proximal humerus, deep to the deltoid muscle, measuring 3.3 × 4.7 × 8.2 cm (AP × TR × CC) (Fig. 1). The mass demonstrates peripheral fat attenuation with internal mineralization which abuts and is intimate with the cortex of the proximal humerus at the greater tuberosity. Differential considerations included osteolipoma, parosteal lipoma, or necrotic changes within a lipoma, although an atypical lipomatous tumor could not be excluded. Follow-up magnetic resonance imaging (MRI) was recommended.

**Fig. 2:**
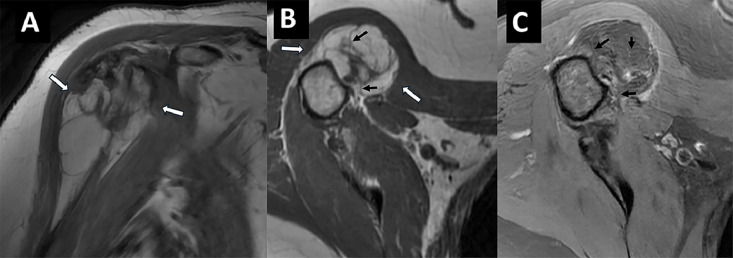
(A) Coronal T1, (B) Axial T1, (C) Axial T2 FS MR sequence of the right shoulder acquired at 3 Tesla with and without intravenous contrast demonstrate predominantly T1 hyperintense lesion at anterior humeral head with thin fibrous septae (black arrows).

MRI performed using tumor protocol demonstrated a predominantly T1 hyperintense lesion following fat signal intensity, measuring 3.0×2.0×2.4 cm (CC × TV × AP), located along the anterolateral humeral head and closely associated with the bicipital groove and biceps tendon (Fig. 2). The lesion was confined beneath the deltoid muscle. Post-contrast images showed no internal central enhancement but did reveal mild peripheral enhancement laterally. The mass demonstrated a lobulated fatty composition with thin, non-enhancing fibrous septa that are low signal on T1 and higher signal on T2 with fat suppression. Foci of calcification were present, appearing as fine linear densities and cortical ossifications, without aggressive features. These findings favored an atypical lipomatous tumor versus a degenerated lipoma with central fat necrosis or a well-differentiated liposarcoma.

Given the indolent nature of the mass, absence of aggressive features, and stable size over a 5-month period, conservative management with observation and interval MRI in 3 = 6 months was recommended, with consideration for biopsy pending further radiographic changes or symptom progression.

## Discussion

Among the differential diagnoses considered, parosteal lipoma is favored based on imaging findings and lesion behavior. The mass is closely adherent to the cortical surface of the proximal humerus, with imaging demonstrating homogeneous fat signal intensity and an associated osseous excrescence, findings which are characteristic of parosteal lipomas [8,10]. Unlike osteolipomas, which are located in soft tissue and lack cortical involvement, this lesion is cortically based [11]. Furthermore, there is no medullary continuity or cartilaginous cap, distinguishing it from osteochondroma [12]. Atypical lipomatous tumors often demonstrate thickened septa, nodularity, or non-fatty enhancing components, none of which were present here [13]. Necrotic lipomas tend to appear heterogeneous or contain fluid levels, which were also absent [14]. Given the lesion’s indolent behavior, stable size over time, and benign imaging features, parosteal lipoma remains the most likely diagnosis. While histological confirmation remains the gold standard, MRI is currently a very valuable tool for evaluating deep lipomatous tumors and guiding management decisions.

A 2021 literature review identified 64 patients reported in case studies and case series with parosteal lipomas, with only 3.1% treated conservatively [15]. However, in many cases, the symptomatic nature of the mass, including associated nerve palsies and muscle atrophy, necessitated surgical excision. Surgical removal of lipomas is typically indicated when the mass exceeds 5 cm in size, is located near neurovascular structures, lies beneath the fascia, causes pain, or presents cosmetic concerns [16]. Interestingly, the median size of excised deep-seated lipomas has been shown to be 11 cm [17]. There is no evidence that parosteal lipomas undergo malignant transformation [4]. With surgical resection, the prognosis is good and local recurrence is rare. Although most parosteal lipomas reported in the literature have been managed surgically due to symptoms or proximity to neurovascular structures, our patient’s lesion was smaller, asymptomatic, and stable in size, with no radiographic signs of malignancy. Given its benign course and negligible risk of malignant transformation, a conservative approach with serial imaging was deemed appropriate.

This case highlights the importance of careful imaging interpretation in differentiating parosteal lipoma from atypical lipomatous tumors and other fat-containing lesions, particularly in deep or anatomically complex regions. MRI remains essential in guiding diagnosis and management, especially when histologic confirmation is deferred.

## Patient consent

Informed written consent was obtained from the patient for publication of this Case Report and all imaging studies. Consent form on record.
